# Construction of Hierarchical Co–Fe Oxyphosphide Microtubes for Electrocatalytic Overall Water Splitting

**DOI:** 10.1002/advs.201900576

**Published:** 2019-07-18

**Authors:** Peng Zhang, Xue Feng Lu, Jianwei Nai, Shuang‐Quan Zang, Xiong Wen (David) Lou

**Affiliations:** ^1^ School of Chemical and Biomedical Engineering Nanyang Technological University 62 Nanyang Drive Singapore 637459 Singapore; ^2^ College of Chemistry and Molecular Engineering Zhengzhou University Henan 450001 P. R. China

**Keywords:** hierarchical, hollow, microtubes, oxyphosphides, water splitting

## Abstract

Development of efficient electrocatalysts is a crucial requirement to build water splitting systems for the production of clean and sustainable fuels. This goal could be achieved by fine‐tuning the composition and structure of the electrocatalytic materials. Here, a facile self‐templated synthetic strategy is developed for the fabrication of hierarchical Co–Fe oxyphosphide microtubes (MTs). Fe‐based metal–organic compound microrods are first synthesized as the self‐sacrificing template. Afterward, the Fe‐based precursors are converted into hierarchical Co–Fe layered double hydroxide MTs through a hydrothermal approach, which are then transformed into the hierarchical Co–Fe oxyphosphide MTs by a phosphidation treatment. Benefiting from the synergistic effect of the compositions and the advantages of the hierarchical hollow structure, the obtained electrocatalyst exhibits enhanced performance for overall water splitting.

## 1 Introduction

Water electrolysis is of great importance for the development of renewable energy sources to solve the energy crisis and environmental problems.^1^, ^2^ Intermittent sustainable energy sources (e.g., solar and wind) can be converted to the versatile and controllable forms through the splitting of water into hydrogen and oxygen.^3^, ^4^, ^5^, ^6^ Recent research has found that Pt‐based materials show outstanding performance for the hydrogen evolution reaction (HER), while RuO_2_ and IrO_2_ are excellent electrocatalysts for the oxygen evolution reaction (OER).^7^ However, the high cost and low abundance of these noble‐metal‐based materials greatly slow down the pace toward practical application of water splitting systems.^8^ Additionally, utilization of different catalysts for HER and OER would further increase the cost due to the complicated process and extra equipment for manufacturing electrodes.^9^, ^10^ Therefore, rational design and fabrication of non‐noble electrocatalysts with high activities toward both OER and HER are still significant and challenging.^11^, ^12^

Apart from the well‐studied metal oxides and (oxy)hydroxides,^9^, ^13^, ^14^, ^15^ transition metal sulfides,^10^, ^16^ phosphides,^17^, ^18^ carbides,^19^, ^20^ and selenides,^21^, ^22^ have been proved as efficient earth‐abundant electrocatalysts toward water splitting. Among these materials, transition metal phosphides have been considered to be one of the most burgeoning categories.^23^ During electrocatalytic water splitting, phosphorus in metal phosphides would be able to participate in the reaction through moderate bonding to the reaction intermediates and create surface with proton acceptor and hydride acceptor sites, leading to high activity.^24^, ^25^ Compared with phosphides with single metal cations, mixed‐metal phosphides exhibit outstanding performance as a result of the synergistic effect of different components.^26^ The introduction of hetero‐metal cations can enhance the transfer of charges between different ions and modify the electronic structure of the material, which would lead to lower kinetic energy barriers for the electrochemical processes.^27^, ^28^ In addition to tuning the cations in phosphide electrocatalysts, doping nonmetal anions is also an effective strategy to promote the electrochemical performance.^29^, ^30^ Therefore, it is believed that efficient electrocatalysts could be constructed by finely manipulating the metal cations and nonmetal anions simultaneously in transition metal phosphides.

Another important approach to enhance the performance of materials for energy related applications is to design advanced structures.^31^, ^32^, ^33^ Among various material architectures, hollow structures show distinct advantages for electrocatalytic water splitting.^34^ Specifically, the large surface areas of hollow nano/micro‐particles can provide abundant active sites for the redox reactions.^35^ Besides, the thin‐shelled topology of hollow structures would promote the transfer of charges and penetration of electrolyte, which are favorable for enhancing the kinetics of water splitting reactions.^36^ Additionally, construction of complex hollow structures with hierarchical morphologies can further increase the surface areas and reduce the charge/mass transfer resistance, which provides new opportunities for the design of highly active electrocatalysts.

## 2 Results and Discussion

**Figure 1:**
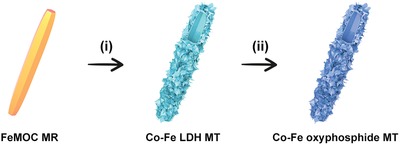
Schematic illustration of the formation process of hierarchical Co–Fe oxyphosphide MTs. i) Conversion of FeMOC MRs into hierarchical Co–Fe LDH MTs through a hydrothermal reaction. ii) Transformation of the hierarchical Co–Fe LDH MTs into hierarchical Co–Fe oxyphosphide MTs by a phosphidation treatment.

Herein, we have developed a facile strategy to fabricate hierarchical Co–Fe oxyphosphide microtubes (MTs) as electrocatalysts for water splitting (**Figure** **1**). First, Fe‐based metal–organic compound (FeMOC) microrods (MRs) are converted into hierarchical Co–Fe layered double hydroxide (LDH) MTs by a hydrothermal reaction (see the Experimental Section for details). Subsequent phosphidation treatment of the as‐prepared LDH MTs leads to the formation of hierarchical Co–Fe oxyphosphide MTs, which exhibit enhanced electrochemical performance for both the OER and HER.

**Figure 2:**
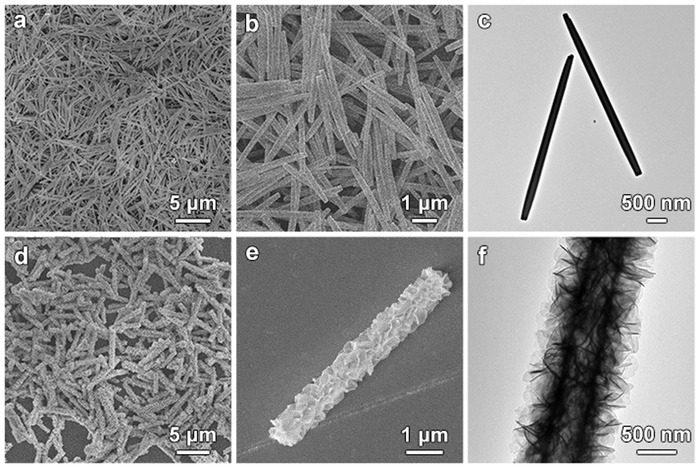
a,b) FESEM and c) TEM images of FeMOC MRs. d,e) FESEM and f) TEM images of hierarchical Co–Fe LDH MTs.

As shown in the field‐emission scanning electron microscopy (FESEM) and transmission electron microscopy (TEM) images, the obtained FeMOC MRs are uniform with the length and diameter of about 4.5 µm and 200 nm, respectively (**Figure** **2**a–c). The sharp peaks in the X‐ray diffraction (XRD) pattern indicate the formation of FeMOC with good crystallinity (Figure S1a, Supporting Information).^37^ Energy‐dispersive X‐ray (EDX) spectrum of FeMOC MRs reveals the existence of Fe, C and O elements, confirming the formation of the FeMOC MRs (Figure S1b, Supporting Information). Subsequently, the as‐prepared FeMOC MRs are converted into Co–Fe LDH MTs through a hydrothermal reaction. Dissolution and outward diffusion of the Fe cations in the alkaline solution lead to the formation of the Co–Fe LDH MTs.^26^ The product possesses a hierarchical structure with nanoflakes grown on the surface (Figure 2d,e). According to the TEM observation, a hollow interior is formed during the reaction (Figure 2f). Diffraction peaks at 10.2°, 20.6°, and 33.9° in the XRD pattern correspond to the characteristic (003), (006), and (012) planes of LDH (Figure S2a, Supporting Information).^38^, ^39^, ^40^, ^41^ EDX spectrum reveals the Co/Fe molar ratio is about 15:1 (Figure S2b, Supporting Information). All these results indicate that hierarchical Co–Fe LDH MTs are successfully synthesized by the hydrothermal conversion of the FeMOC MR precursors.

**Figure 3:**
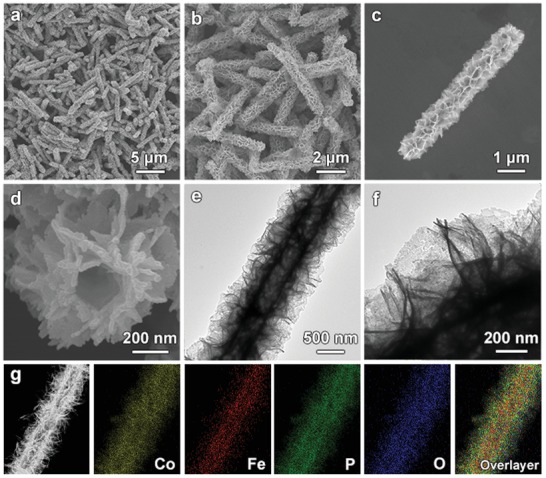
a–d) FESEM and e,f) TEM images of hierarchical Co–Fe oxyphosphide MTs. g) HAADF‐STEM image of a hierarchical Co–Fe oxyphosphide MT with elemental mappings for Co, Fe, P, O, and corresponding overlay image.

Phosphidation treatment is then performed through the reaction of Co–Fe LDH MTs with PH_3_ gas generated from the thermal decomposition of NaH_2_PO_2_. XRD pattern of the sample can be indexed to Co–Fe phosphide‐based materials (Figure S3a, Supporting Information).^42^, ^43^ Besides, EDX spectrum indicates the product possesses a similar Co/Fe molar ratio as that of the Co–Fe LDH MTs (Figure S3b, Supporting Information). The sample also contains a large amount of O element with the O/P molar ratio of about 1:1.5.^44^ High‐resolution TEM (HRTEM) observation shows the lattice fringes with spacings of 0.243 and 0.252 nm, corresponding to the (102) planes of the CoP and FeP, respectively (Figure S4, Supporting Information). Moreover, X‐ray photoelectron spectroscopy (XPS) analysis of the sample indicates the formation of P—O and M—O bonds during the phosphidation process (Figure S5, Supporting Information). These results suggest that Co–Fe oxyphosphide is formed after the phosphidation reaction. As shown in the FESEM images (**Figure** **3**a–c), the hierarchical structure with nanoflakes on the surface is preserved. FESEM image of one broken particle manifests the tubular structure of the sample (Figure 3d), which is further confirmed by the TEM observation (Figure 3e). TEM image at a higher magnification reveals the ultrathin nature of the nanoflakes on the surface (Figure 3f). High‐angle annular dark‐field scanning transmission electron microscopy (HAADF‐STEM) and elemental mapping images further prove the formation of the hierarchical Co–Fe oxyphosphide MTs (Figure 3g).

**Figure 4:**
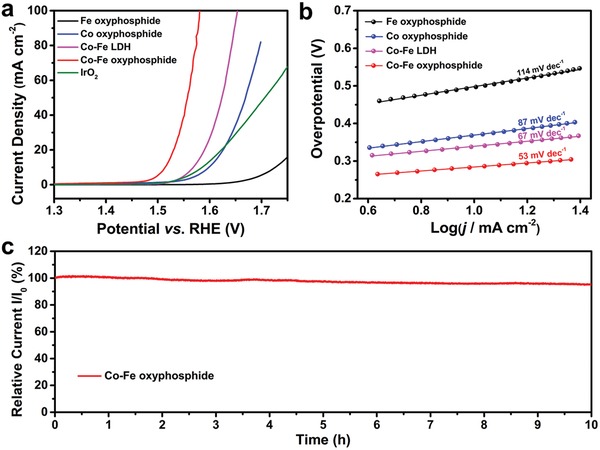
a) LSV curves of Fe oxyphosphide, Co oxyphosphide, Co–Fe LDH, Co–Fe oxyphosphide, and benchmark IrO_2_ for OER. b) Tafel plots of Fe oxyphosphide, Co oxyphosphide, Co–Fe LDH, and Co–Fe oxyphosphide for OER. c) Time‐dependent curve of relative current density to the initial 10 mA cm^−2^ of Co–Fe oxyphosphide for OER.

In order to reveal the advantages of the Co–Fe oxyphosphide MTs, control samples with single metal cations are fabricated. Hierarchical Co oxyphosphide microspheres (MSs) are synthesized through a similar sequential hydrothermal and phosphidation approach without the inducing of FeMOC precursors (Figure S6–S8, Supporting Information). Besides, Fe oxyphosphide MRs are obtained by direct phosphidation of the FeMOC MRs (Figures S9 and S10, Supporting Information). The electrocatalytic performance for OER is evaluated in an alkaline solution (1.0 m KOH) using a standard three‐electrode cell. **Figure** **4**a shows the linear sweep voltammetry (LSV) curves of different samples. It can be observed that the Co–Fe oxyphosphide MTs exhibit a lower onset potential and a higher current density for the OER than the other control samples as well as the benchmark IrO_2_ electrocatalyst. At the same time, the current density of 10 mA cm^−2^ (a common criterion to evaluate the electrocatalytic activity) can be reached by the Co–Fe oxyphosphide MTs at the potential of 1.51 V versus reversible hydrogen electrode (RHE), corresponding to an overpotential of 280 mV. Nevertheless, higher overpotentials of 340, 370, and 500 mV are required for Co–Fe LDH MTs, Co oxyphosphide MSs, and Fe oxyphosphide MRs, respectively, to achieve the same current density. The low overpotential of the hierarchical Co–Fe oxyphosphide MTs compares favorably to those of representative phosphide‐based electrocatalysts (Table S1, Supporting Information). Additionally, the mass activity of Co–Fe oxyphosphide MTs at the overpotential of 300 mV for OER is also higher than that of the control samples (Figure S11a, Supporting Information). Tafel plots of different electrocatalysts are collected based on the LSV curves (Figure 4b). The linear regions in the Tafel plots are fitted to the Tafel equation (η = *b*log*j* + *a*, where η is the overpotential, *j* is the current density, and *b* is the Tafel slope). The Co–Fe oxyphosphide MTs exhibit enhanced electrochemical performance for OER as revealed by the lower Tafel slop of 53 mV dec^−1^ compared with those of the control samples.^10^ The long‐term stability of the Co–Fe oxyphosphide MTs is also tested. About 95% of the initial current density is retained after 10 h of reaction (Figure 4c). All these results demonstrate the high activity and stability of the hierarchical Co–Fe oxyphosphide MTs toward OER.

**Figure 5:**
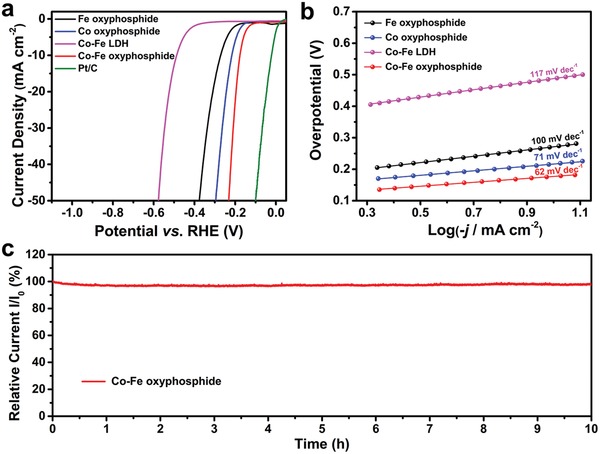
a) LSV curves of Fe oxyphosphide, Co oxyphosphide, Co–Fe LDH, Co–Fe oxyphosphide, and benchmark Pt/C for HER. b) Tafel plots of Fe oxyphosphide, Co oxyphosphide, Co–Fe LDH, and Co–Fe oxyphosphide for HER. c) Time‐dependent curve of relative current density to the initial −10 mA cm^−2^ of Co–Fe oxyphosphide for HER.

The electrocatalytic performance for HER is also evaluated in the same alkaline medium and three‐electrode cell. As observed from the LSV curves of different samples, the Co–Fe oxyphosphide MTs exhibit an overpotential of about 180 mV to reach the current density of −10 mA cm^−2^ (**Figure** **5**a). Larger overpotentials of 220, 270, and 490 mV are required for Co oxyphosphide MSs, Fe oxyphosphide MRs, and Co–Fe LDH MTs, respectively. However, the performance of the Co–Fe oxyphosphide MTs is not as good as the benchmark Pt/C electrocatalyst. The mass activity of Co–Fe oxyphosphide MTs toward HER at the overpotential of 200 mV is higher than that of the control samples (Figure S11b, Supporting Information). At the same time, the Co–Fe oxyphosphide MTs exhibit the lowest Tafel slope of 62 mV dec^−1^ for HER among all the samples, implying the favorable hydrogen evolution (Figure 5b).^24^ Moreover, about 98% of the initial current density is preserved after the stability test, revealing the good stability of the Co–Fe oxyphosphide MTs toward HER (Figure 5c). As indicated by cyclic voltammogram (CV) curves (Figure S12, Supporting Information) and corresponding capacitive current plots against the scan rate (Figure S13, Supporting Information), the Co–Fe oxyphosphide MTs possess the largest double‐layer capacitance (*C*_dl_) among the samples. This result indicates that the Co–Fe oxyphosphide MTs have relatively large electrochemically active surface area (ECSA), which would result in the promoted electrocatalytic performance. The ECSA‐normalized LSV curves of different samples show similar trends as the geometric area‐normalized ones for the OER and HER, indicating the intrinsic high electrocatalytic activity of the Co–Fe oxyphosphide MTs (Figure S14, Supporting Information). Moreover, the low charge‐transfer resistance of the Co–Fe oxyphosphide MTs, as characterized by the electrochemical impedance spectroscopy (EIS), might be conducive to the transfer of electrons during the electrocatalytic reactions, leading to the high activity (Figure S15, Supporting Information). These results suggest that the hierarchical hollow structure, the synergetic effect of the bimetallic cations and the formation of oxyphosphides could enhance the activity of the Co–Fe oxyphosphide MTs toward electrocatalytic water splitting.

To evaluate the electrocatalytic performance of the hierarchical Co–Fe oxyphosphide MTs for overall water splitting, an alkaline (1.0 m KOH) electrolyzer was constructed with a two‐electrode configuration. The current density of 10 mA cm^−2^ is reached at the bias of 1.69 V (Figure S16, Supporting Information). Besides, the electrolyzer shows good stability with about 93% of the initial activity retained after the stability test of 10 h (Figure S17, Supporting Information). The FESEM and TEM images of the Co–Fe oxyphosphide MTs on the anode of the electrolyzer after the stability test indicate that the hierarchical tubular structure is preserved (Figure S18a,c, Supporting Information). However, the sharp edges of the nanoflakes become roundish, which might be induced by the transformation of the oxyphosphides on the surface into amorphous hydroxides during the OER.^45^ This hypothesis can be proved by the HRTEM observation, which shows the formation of the surface amorphous layer (Figure S19a, Supporting Information). Furthermore, the content of P element in the sample drops greatly after the long‐term OER Figure S20a Supporting Information). At the same time, Co^2+^ at the surface is oxidized to Co^3+^ with the formation of M—O—OH bonds (Figure S21, Supporting Information). These results suggest that an amorphous layer of metal hydroxides is formed on the anode of the electrolyzer, which acts as the active component for OER. On the contrary, no obvious morphological change is found for the Co–Fe oxyphosphide MTs on the cathode (Figure S18b,d, Supporting Information), indicating the good structural stability of the electrocatalyst during the HER. Meanwhile, the composition and surface properties of the Co–Fe oxyphosphide MTs are well maintained (Figures S19b, S20b, and S22, Supporting Information). The XRD patterns of the Co–Fe oxyphosphide MTs after the stability test reveal that the bulk of the electrocatalysts is not changed during the reactions (Figure S23, Supporting Information). The electrochemical performance of the hierarchical Co–Fe oxyphosphide MTs toward overall water splitting is comparable with the previously reported results (Table S1, Supporting Information).

## 3 Conclusions

In summary, hierarchical Co–Fe oxyphosphide microtubes have been fabricated through a facile self‐templated synthetic strategy. First, Fe‐based metal–organic compound microrods are converted into hierarchical Co–Fe layered double hydroxides microtubes via a hydrothermal reaction. A subsequent phosphidation process leads to the formation of the hierarchical Co–Fe oxyphosphide microtubes. The obtained electrocatalyst exhibits good performance in alkaline electrolyte for both the oxygen evolution reaction and the hydrogen evolution reaction, with the overpotentials of 280 and 180 mV, respectively, to reach a current density of 10 mA cm^−2^. In addition, the material is relatively stable for long‐term operations. The developed approach may inspire further capability on the construction of hierarchical hollow‐structured materials for electrocatalytic water splitting and other energy‐related applications.

## 4 Experimental Section

*Synthesis of FeMOC MRs*: 20 mg of iron(II) acetate and 40 mg of polyvinylpyrrolidone (K30) were dissolved in 0.75 mL of ethylene glycol under stirring. Then, 2.25 mL of methanol was added. The solution was transferred to a Teflon‐lined stainless steel autoclave and kept at 120 °C for 12 h. The products were collected by centrifugation and washed with ethanol three times, which were then dissolved in 10 mL of ethanol.

*Synthesis of Hierarchical Co–Fe LDH MTs*: 1 mL of the obtained solution of FeMOC MRs was added into 5 mL of 50 × 10^−3^ m Co(NO_3_)_2_ · 6H_2_O methanol solution. Subsequently, 5 mL of 50 × 10^−3^ m 2‐methylimidazole methanol solution was added. The solution was transferred to a Teflon‐lined stainless steel autoclave and kept at 120 °C for 4 h. The products were collected by centrifuge and washed with ethanol three times, which were then dried in an oven at 70 °C.

*Synthesis of Hierarchical Co–Fe Oxyphosphide MTs*: The obtained Co–Fe LDH MTs were annealed with NaH_2_PO_2_ at 350 °C for 2 h with a ramp rate of 5 °C min^−1^ under a flow of nitrogen gas.

*Synthesis of Hierarchical Co Oxyphosphide MSs*: First, 5 mL of 50 × 10^−3^ m 2‐methylimidazole methanol solution was added into 5 mL of 50 × 10^−3^ m Co(NO_3_)_2_ · 6H_2_O methanol solution. The obtained solution was then transferred to a Teflon‐lined stainless steel autoclave and kept at 120 °C for 4 h. The products were collected by centrifuge and washed with ethanol three times, which were then dried in an oven at 70 °C. Second, the obtained hierarchical Co‐Co LDH MSs were annealed with NaH_2_PO_2_ at 350 °C for 2 h with a ramp rate of 5 °C min^−1^ under a flow of nitrogen gas.

*Synthesis of Fe Oxyphosphide MRs*: FeMOC MRs were annealed with NaH_2_PO_2_ at 350 °C for 2 h with a ramp rate of 5 °C min^−1^ under a flow of nitrogen gas.

*Materials Characterization*: The crystal phase was examined by X‐ray diffraction (XRD) on a Bruker D2 Phaser X‐Ray Diffractometer. Field‐emission scanning electron microscope (FESEM; JEOL‐6700F) and transmission electron microscope (TEM; JEOL, JEM‐2010) were used to characterize the morphology and structure. The composition was analyzed by EDX spectroscopy. Surface chemical analysis was performed by X‐ray photoelectron spectroscopy (XPS, ESCALAB 250Xi) with Al Kα beam source.

*Electrochemical Measurements*: For all the electrochemical measurements, an aqueous solution of 1.0 m KOH was used as the electrolyte. Electrochemical measurements were evaluated in a three‐electrode configuration using a rotating disk electrode (RDE, PINE Research Instrumentation) at a rotation speed of 1600 rpm with a CHI 660D electrochemical workstation. A glassy carbon (GC) disk electrode (5 mm in diameter) was used as the working electrode. The catalyst suspension was prepared by dispersing 5 mg of catalyst in 1 mL of solution containing 0.95 mL of ethanol and 50 µL of 0.5 wt% nafion solution followed by ultrasonication for 30 min. Then, 10 µL of the above suspension was dropped on the polished GC electrode and then dried at room temperature. Carbon rod and Ag/AgCl (KCl saturated) electrodes were used as the counter electrode and reference electrode, respectively. Potentials were referenced to a RHE: *E*_RHE_ = *E*_Ag/AgCl_ + 0.197 + 0.059 × pH. LSV curves were collected with *iR* drop compensated. The electrochemically active surface area (ECSA) of the catalyst was calculated by measuring the double‐layer capacitance (*C*_dl_) under a potential window from −0.1 to 0 V versus Ag/AgCl. The ECSA was obtained by dividing the *C*_dl_ by a general specific capacitance (*C*_s_) of 0.04 mF cm^−2^.^46^ Electrochemical impedance spectroscopy (EIS) measurements were conducted in the range of 0.1–10^5^ Hz with 0.01 V amplitude. For stability tests and overall water splitting tests, the work electrodes were prepared by dropping 10 uL of the prepared catalyst suspension onto carbon paper with an active surface area of 0.25 cm^2^. Overall water splitting tests were conducted in a two‐electrode configuration with *iR* drop compensated.

## Conflict of Interest

The authors declare no conflict of interest.

## Supporting information

Supplementary
