# Supplementary material for: Construction of Hierarchical Co–Fe Oxyphosphide Microtubes for Electrocatalytic Overall Water Splitting
Source: Adv Sci (Weinh). 2019 Jul 18;6(17):1900576. doi: 10.1002/advs.201900576 (PMC6724352; doi:10.1002/advs.201900576)
Supplement: Supplementary file 1 — Supplementary [file ADVS-6-1900576-s001.pdf]

Copyright WILEY-VCH Verlag GmbH & Co. KGaA, 69469 Weinheim, Germany, 2019.

ADVANCED SCIENCE Open Access

Supporting Information

for *Adv. Sci.,* DOI: 10.1002/advs.201900576

Construction of Hierarchical Co–Fe Oxyphosphide Microtubes for Electrocatalytic Overall Water Splitting

Peng Zhang, Xue Feng Lu, Jianwei Nai, Shuang-Quan Zang, and Xiong Wen (David) Lou**

Supporting Information *for*

Construction of Hierarchical Co-Fe Oxyphosphide Microtubes for Electrocatalytic Overall Water Splitting

Peng Zhang, Xue Feng Lu, Jianwei Nai, Shuang-Quan Zang, and Xiong Wen (David) Lou**

[*] Dr. P. Zhang, Dr. X. F. Lu, Dr. J. W. Nai, Prof. X. W. Lou

School of Chemical and Biomedical Engineering, Nanyang Technological University, 62 Nanyang Drive, Singapore, 637459, (Singapore)

Email: xwlou@ntu.edu.sg (X.W.L.) or jwnai@zjut.edu.cn (J.W.N.)

Webpage: http://www.ntu.edu.sg/home/xwlou/

Prof. S. Q. Zang

College of Chemistry and Molecular Engineering, Zhengzhou University, Henan 450001, P. R. China

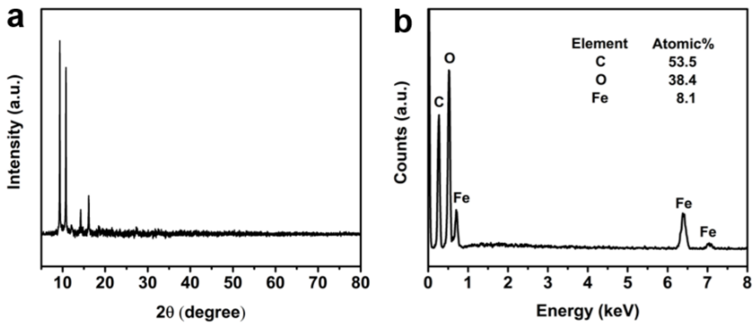

Figure S1. (a) XRD pattern and (b) EDX spectrum of FeMOC MRs.

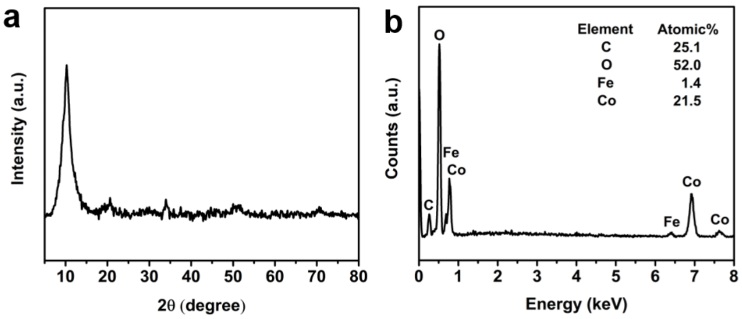

Figure S2. (a) XRD pattern and (b) EDX spectrum of hierarchical Co-Fe LDH MTs.

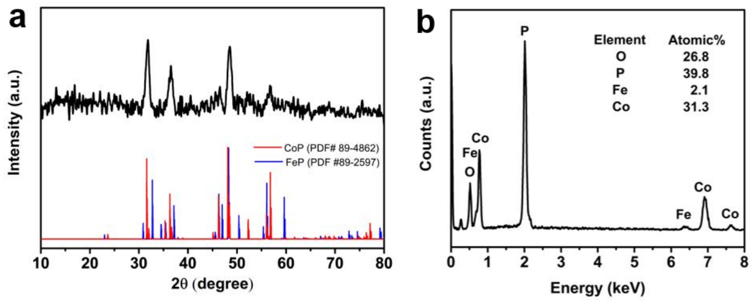

Figure S3. (a) XRD pattern and (b) EDX spectrum of hierarchical Co-Fe oxyphosphide MTs.

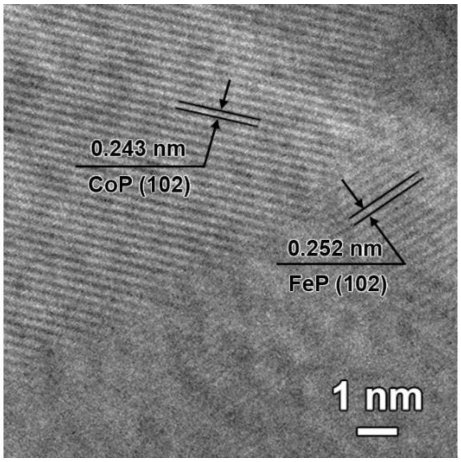

Figure S4. HRTEM image of hierarchical Co-Fe oxyphosphide MTs.

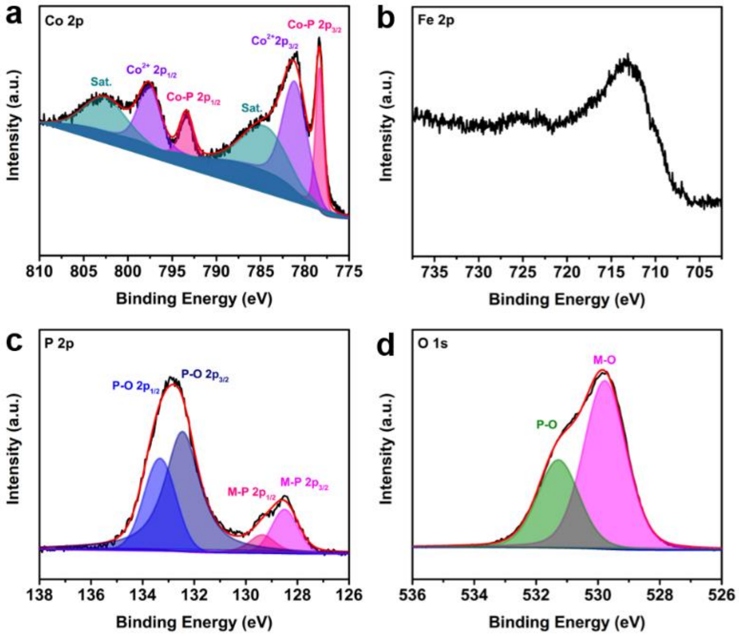

Figure S5. XPS spectra for (a) Co 2p, (b) Fe 2p, (c) P 2p and (d) O 1s of hierarchical Co-Fe oxyphosphide MTs.

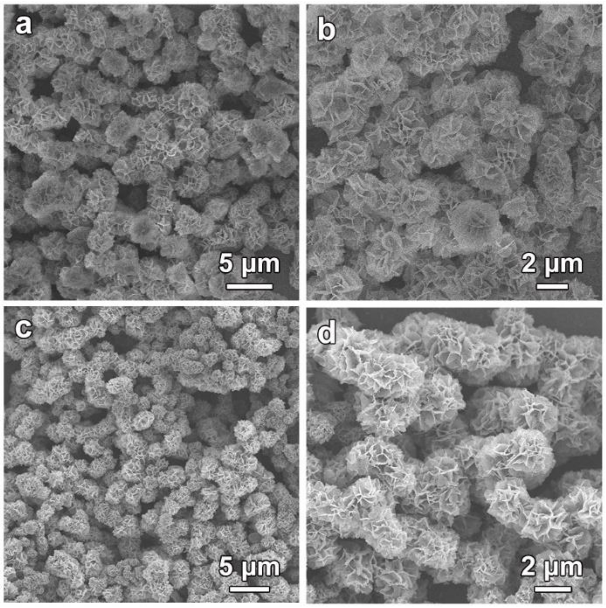

Figure S6. FESEM images of (a,b) hierarchical Co-Co LDH MSs and (c,d) hierarchical Co oxyphosphide MSs.

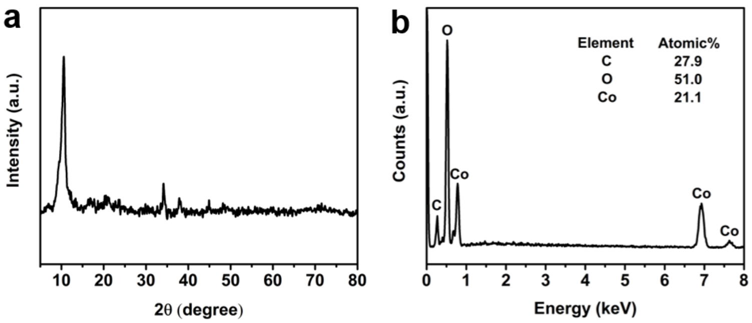

Figure S7. (a) XRD pattern and (b) EDX spectrum of hierarchical Co-Co LDH MTs.

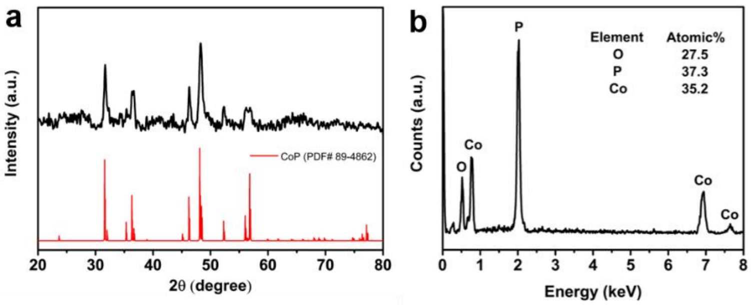

Figure S8. (a) XRD pattern and (b) EDX spectrum of hierarchical Co oxyphosphide MTs.

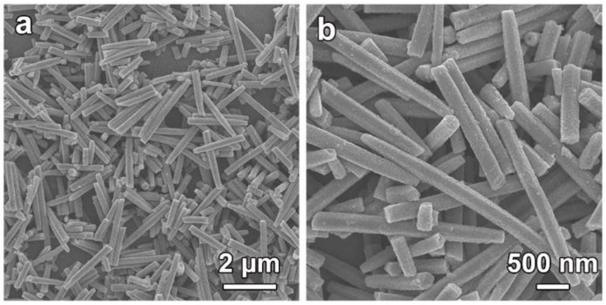

Figure S9. FESEM images of Fe oxyphosphide MRs.

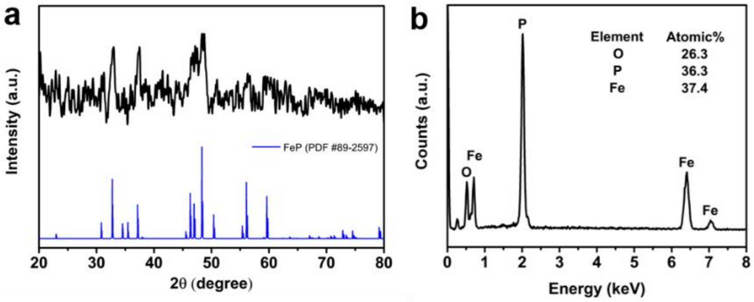

Figure S10. (a) XRD pattern and (b) EDX spectrum of Fe oxyphosphide MRs.

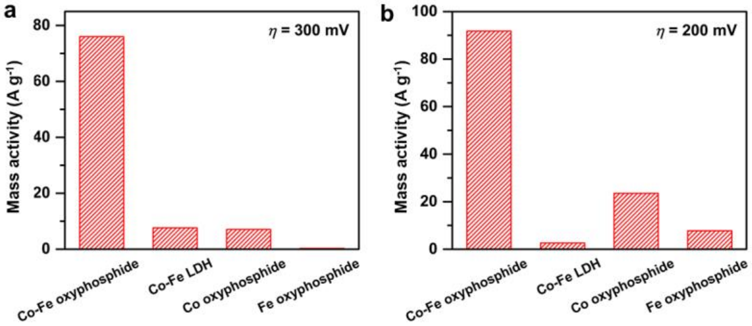

Figure S11. Mass activities of Fe oxyphosphide, Co oxyphosphide, Co-Fe LDH and Co-Fe oxyphosphide at overpotentials of 300 mV and 200 mV for (a) OER and (b) HER.

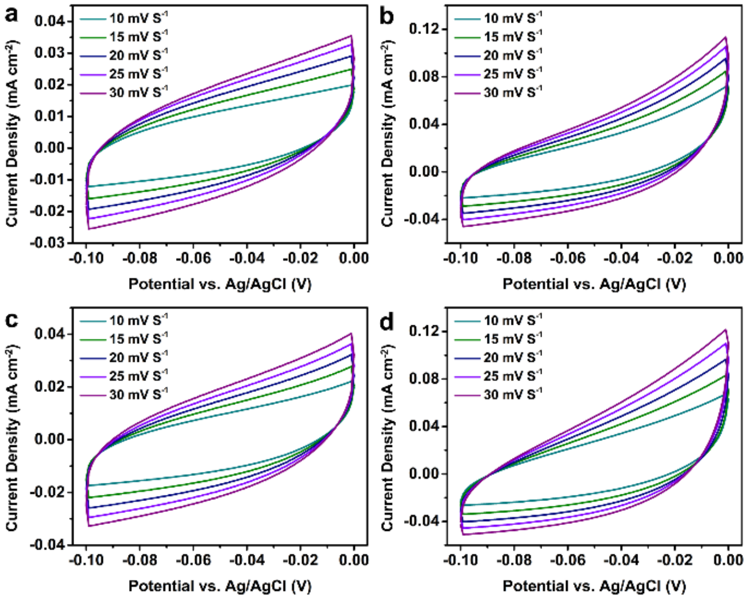

Figure S12. CV curves at different scan rates for (a) Fe oxyphosphide, (b) Co oxyphosphide, (c) Co-Fe LDH, and (d) Co-Fe oxyphosphide.

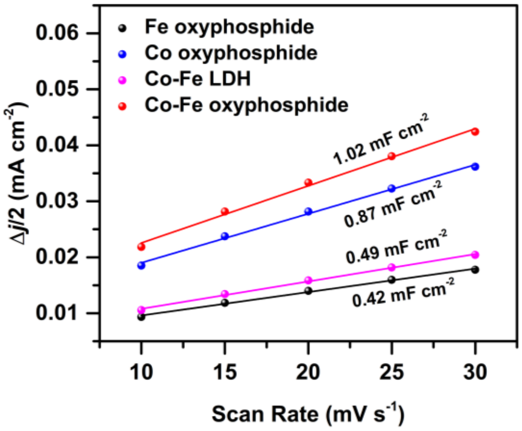

Figure S13. Capacitive current as a function of scan rate for Fe oxyphosphide, Co oxyphosphide, Co-Fe LDH, and Co-Fe oxyphosphide.

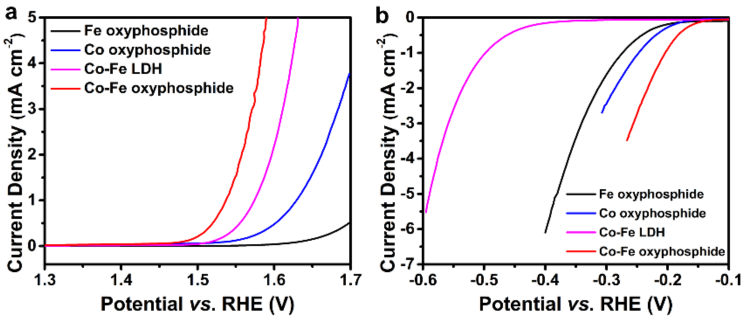

Figure S14. ECSA-normalized LSV curves of Fe oxyphosphide, Co oxyphosphide, Co-Fe LDH, and Co-Fe oxyphosphide for (a) OER and (b) HER.

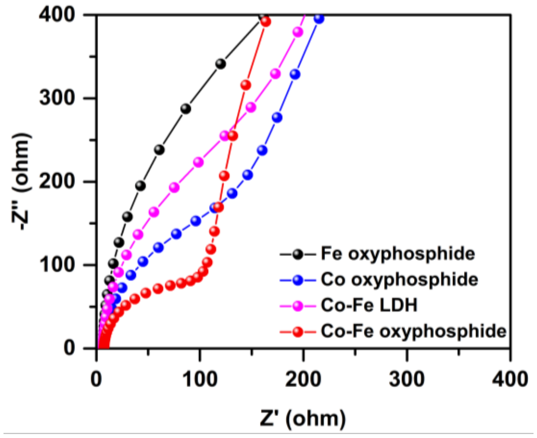

Figure S15. Nyquist plots of Fe oxyphosphide, Co oxyphosphide, Co-Fe LDH, and Co-Fe oxyphosphide.

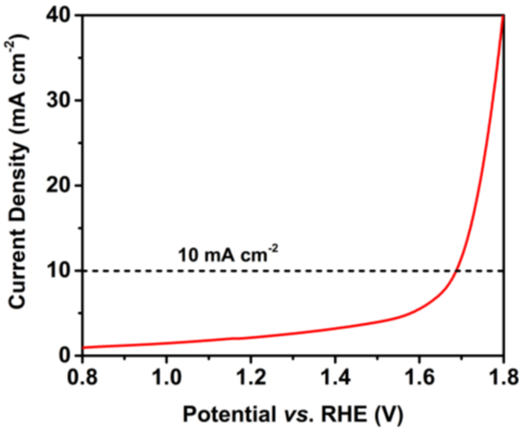

Figure S16. LSV curve of the hierarchical Co-Fe oxyphosphide MTs(±) electrolyzer.

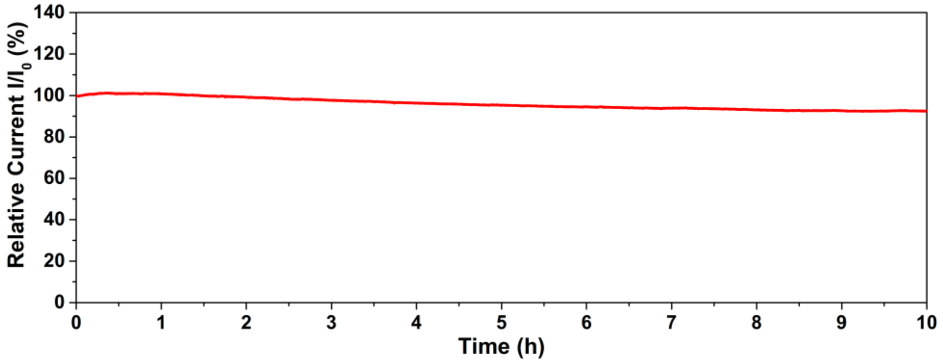

Figure S17. Time-dependent curve of relative current density to the initial 10 mA cm^{-2} of the hierarchical Co-Fe oxyphosphide MTs(±) electrolyzer.

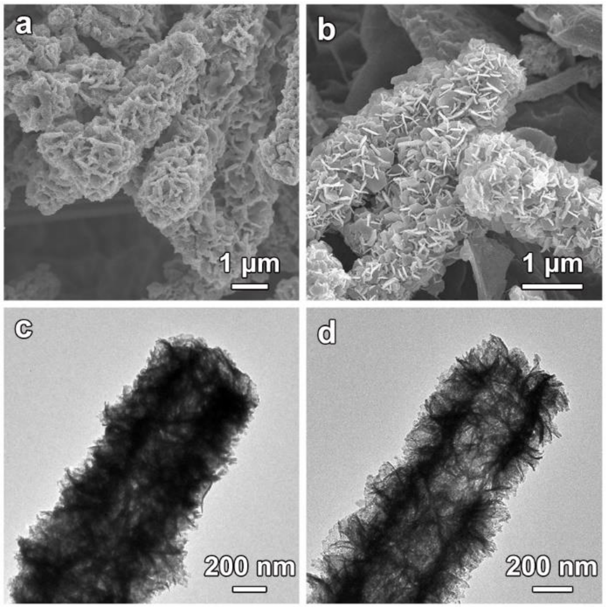

Figure S18. (a,b) FESEM and (c,d) TEM images of Co-Fe oxyphosphide MTs on the anode (a,c) and cathode (b,d) of the electrolyzer after the stability test.

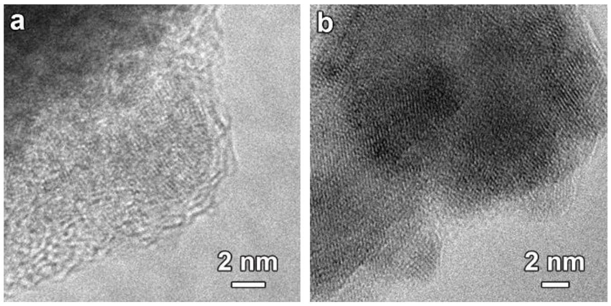

Figure S19. HRTEM images of Co-Fe oxyphosphide MTs on the (a) anode and (b) cathode of the electrolyzer after the stability test.

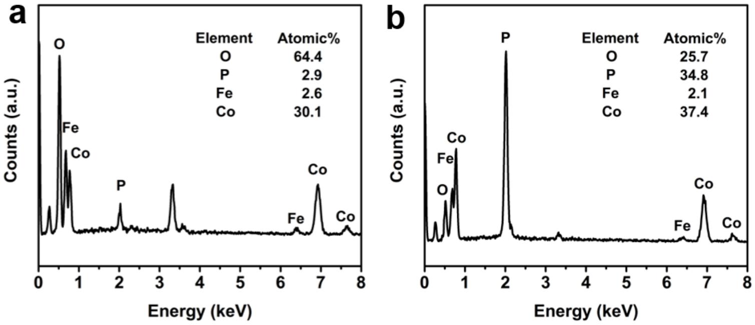

Figure S20. EDX spectra of Co-Fe oxyphosphide MTs on the (a) anode and (b) cathode of the electrolyzer after the stability test.

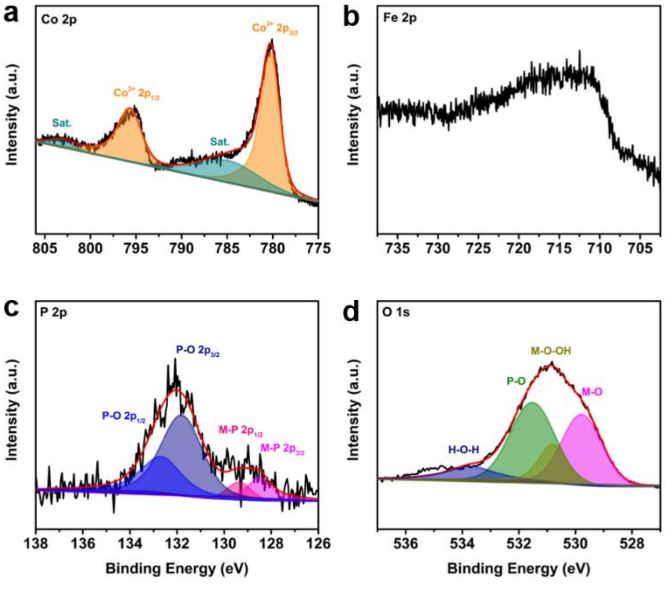

Figure S21. XPS spectra for (a) Co 2p, (b) Fe 2p, (c) P 2p and (d) O 1s of hierarchical Co-Fe oxyphosphide MTs on the anode of the electrolyzer after the stability test.

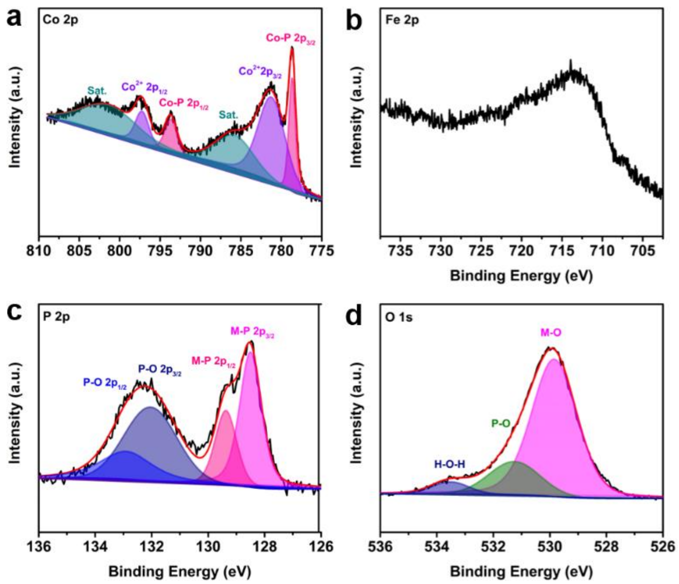

Figure S22. XPS spectra for (a) Co 2p, (b) Fe 2p, (c) P 2p and (d) O 1s of hierarchical Co-Fe oxyphosphide MTs on the cathode of the electrolyzer after the stability test.

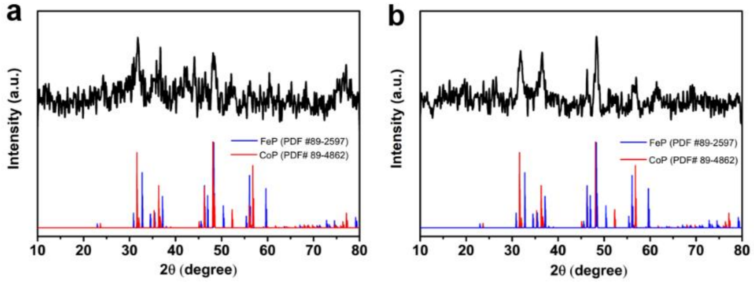

Figure S23. XRD patterns of Co-Fe oxyphosphide MTs on the (a) anode and (b) cathode of the electrolyzer after the stability test.

Table S1. Summary of the electrocatalytic performance of different electrocatalysts reported recently for overall water splitting.

Catalyst	Electrolyte	η for HER (mV)[a]	η for OER (mV)[b]	Voltage for overall water splitting (V)[b]	Ref.
Co-Fe oxyphosphide	1 M KOH	180	280	1.69	This work
Co-Fe-P foam	1 M KOH	73	294	N.A.	[1]
Co_xFe_{1-x}-P film	1 M KOH	169	290	1.64	[2]
Fe-Co-Pi-Bi/CC	0.1 M K-Bi	175	382	1.95	[3]
$Co_{0.68}Fe_{0.32}P$	1 M KOH	116	289	N.A.	[4]
CoP/rGO	1 M KOH	150	340	1.70	[5]
CoP_2/rGO	1 M KOH	115	370	1.68	[6]
S:CoP/Ni foam	1 M KOH	109	270	~1.60	[7]
CoP film/Cu foil	1 M KOH	94	345	~1.69	[8]
FeP@Fe-P-O/CC	1 M KOH	120	288	1.69	[9]
FeP/Ni foam	1 M KOH	166	227	1.59	[10]

[a] corresponding data were obtained with a current density of -10 mA cm^{-2}

[b] corresponding data were obtained with a current density of 10 mA cm^{-2}

Supplementary References:

[1] H. Kim, S. Oh, E. Cho, H. Kwon, *ACS Sustainable Chem. Eng.* **2018**, *6*, 6305.

[2] S. Yoon, J. Kim, J.-H. Lim, B. Yoo, *J. Electrochem. Soc.* **2018**, *165*, H271.

[3] M. Ma, G. Zhu, F. Xie, F. Qu, Z. Liu, G. Du, A. M. Asiri, Y. Yao, X. Sun, *ChemSusChem* **2017**, *10*, 3188.

[4] F. Li, Y. Bu, Z. Lv, J. Mahmood, G.-F. Han, I. Ahmad, G. Kim, Q. Zhong, J.-B. Baek, *Small* **2017**, *13*, 1701167.

[5] L. Jiao, Y.-X. Zhou, H.-L. Jiang, *Chem. Sci.* **2016**, *7*, 1690.

[6] J. Wang, W. Yang, J. Liu, *J. Mater. Chem. A* **2016**, *4*, 4686.

[7] M. A. R. Anjum, M. S. Okyay, M. Kim, M. H. Lee, N. Park, J. S. Lee, *Nano Energy* **2018**, *53*, 286.

[8] N. Jiang, B. You, M. Sheng, Y. Sun, *Angew. Chem. Int. Ed.* **2015**, *54*, 6251.

[9] Y. Yan, B. Y. Xia, X. Ge, Z. Liu, A. Fisher, X. Wang, *Chem. Eur. J.* **2015**, *21*, 18062.

[10] S. Yao, V. Forstner, P. W. Menezes, C. Panda, S. Mebs, E. M. Zolnhofer, M. E. Miehlich, T. Szilvási, N. Ashok Kumar, M. Haumann, K. Meyer, H. Grützmacher, M. Driess, *Chem. Sci.* **2018**, *9*, 8590.
